# Isolation of Various Flavonoids with TRAIL Resistance-Overcoming Activity from *Blumea lacera*

**DOI:** 10.3390/molecules28010264

**Published:** 2022-12-28

**Authors:** Teruhisa Manome, Yasumasa Hara, Masami Ishibashi

**Affiliations:** 1 Graduate School of Pharmaceutical Sciences, Chiba University, 1-8-1 Inohana, Chuo-ku, Chiba 260-8675, Japan; 2 Plant Molecular Science Center, Chiba University, 1-8-1 Inohana, Chuo-ku, Chiba 260-8675, Japan

**Keywords:** flavonoids, *Blumea lacera*, TRAIL resistance, Thai plant

## Abstract

Eighteen compounds, including fourteen flavonoids (**1**–**14**), one steroid (**15**), two fatty acids (**16**,**17**), and one nitrogen-containing compound (**18**), were isolated from the methanol extract of the whole *Blumea lacera* plant collected in Thailand. Compounds **1**–**11** and **15**–**17** exhibited tumor necrosis factor-related apoptosis-inducing ligand (TRAIL) resistance-overcoming activity. Among them, bonanzin (**2**) and cirsilineol (**7**) had particularly strong TRAIL resistance-overcoming activity, where the IC_50_ values against the human gastric adenocarcinoma cell line AGS in the presence of TRAIL (100 ng/mL) were 10.7 μM and 5.9 μM, respectively.

## 1. Introduction

Tumor necrosis factor-related apoptosis-inducing ligand (TRAIL) binds to the death receptor and selectively induces apoptosis in cancer cells. Therefore, TRAIL is considered an attractive therapeutic target for cancer treatment. However, various types of cancer cells, including gastric, prostate, and breast cancers, are resistant to TRAIL-induced apoptosis [1]. Thus, compounds that can overcome this resistance may lead to the development of new anticancer drugs.

*Blumea lacera* is an annual herb in the Asteraceae family that is distributed in Southeast Asia, Australia, China, India, and tropical Africa. This plant has been used as traditional medicine by exploiting its antipyretic, antioxidant, and stimulant [2] properties, and is reported to have antiviral [3], anti-leukemic [3], and cytotoxic activities against several cancer cell lines [4,5]. Several compounds such as nicotifiorin [5], mauritianin [5], and rotundioic acid [6] have been isolated from *Blumea lacera*, whereas the bioactive natural compounds with TRAIL resistance-overcoming activity have not been explored in this plant.

We previously reported some naturally derived compounds with TRAIL resistance-overcoming activity [7,8]. In this study, we describe the isolation of eighteen compounds from the whole plant of *Blumea lacera* and report their TRAIL resistance-overcoming activity.

## 2. Materials and Methods

### 2.1. General Experimental Procedures

The following instruments were used in this study: a P-2200 (JASCO, Hachioji, Japan) polarimeter for measuring optical rotation, ECZ600 (JEOL, Akishima, Japan) instrument for NMR spectroscopy (solvent chemical shifts were used as internal standards), PU2080 Plus pump, UV 2075, and RI-2031 Plus (JASCO, Hachioji, Japan) detectors for HPLC. An additional HPLC system comprising an LC-20AB pump, CTO-20AC column oven (40 °C), and SPD-M20A UV-vis photodiode array detector (λ = 190–800 nm, Shimadzu, Kyoto, Japan) was also used.

The following adsorbents were used for purification: silica gel 60 F_254_ (0.25 mm), 60 RP-18 F_254S_ (0.25 mm), and silica gel 60 DiolF_254S_ (0.25 mm, Merck, Darmstadt, Federal Republic of Germany) for analytical thin-layer chromatography (TLC); Chromatorex ODS (Fuji Silysia Chemical, Ltd., Kasugai, Japan), silica gel 60 N (Kanto Chemical Co., Inc., Tokyo, Japan), and Sephadex LH-20 (GE Healthcare, Chicago, USA) for column chromatography; and COSMOSIL Cholester (*ϕ* 10.0 × 250 mm) and COSMOSIL πNAP (*ϕ* 10.0 × 250 mm, Nacalai Tesque Inc., Kyoto, Japan), and Inertsil C8-3 5μm (*ϕ* 10.0 × 250 mm, GL Sciences, Tokyo, Japan) for preparative HPLC.

### 2.2. Plant Material

Whole plants of *Blumea lacera* (Asteraceae) were collected from Thailand and identified by the late Thaworn Kowithayakorn. A voucher specimen (KKP649) was deposited in the Department of Natural Products Chemistry, Graduate School of Pharmaceutical Sciences, Chiba University, Japan.

### 2.3. Cell Cultures

AGS cells were purchased from ATCC and cultured in Roswell Park Memorial Institute 1640 medium (Wako, Osaka, Japan) supplemented with 10% fetal bovine serum (FBS, Biowest, Nuaillé, France) and 1% penicillin-streptomycin (PS, Sigma, St. Louis, MO, USA). Cultures were maintained in a humidified incubator at 37 °C in CO_2_/air (5/95).

### 2.4. Assay of Cell Viability (TRAIL Resistance-Overcoming Activity Assay)

Cell viability was assessed using a fluorometric microculture cytotoxicity assay [9] in the presence and absence of TRAIL using TRAIL-resistant AGS cells. Cells were seeded in 96-well culture plates (1 × 10^4^ cells per well) in 200 μL of medium containing 10% FBS and 1% PS. After 24 h of incubation, samples with or without TRAIL (100 ng/mL) were added to each well. Dimethyl sulfoxide (DMSO, 0.1%) was used as the negative control. Luteolin (17.5 μM) was used as a positive control. After another 24 h of incubation, the cells were washed with phosphate-buffered saline (PBS), and 200 μL PBS containing fluorescein diacetate (10 μg/mL) was added to each well. The plates were incubated at 37 °C for 1 h, and fluorescence at 538 nm with excitation at 485 nm was measured using a Fluoroskan Ascent (Thermo Fisher Scientific, Waltham, USA). IC_50_ values were calculated using TRAIL (0 ng/mL), and the survival rate was 100%. For example, the IC_50_ value of **2** with TRAIL was determined based on the viability at 7 µM (85.9%) and 13 µM (33.9%).

### 2.5. Extraction and Isolation

Dried and ground whole *Blumea lacera* plants (81 g) were extracted with MeOH overnight at room temperature four times to obtain a crude MeOH extract (7.2 g). The MeOH extract (7.2 g) was partitioned with hexane, CHCl_3_, EtOAc, and BuOH to obtain the respective layers. The hexane layer (1.4 g) was fractionated using silica gel 60N column chromatography (*ϕ* 40 × 200 mm, hexane/EtOAc, and CHCl_3_/MeOH system) to obtain fractions 1A–1R. Fraction 1D (hexane/EtOAc = 10/1, 43.2 mg) was separated using reverse-phase (RP) HPLC (Inertsil C8-3 5 μm, *ϕ* 10 × 250 mm; 95% acetonitrile (CH_3_CN); flow rate 5.0 mL/min) to isolate compound **15** (1.7 mg, *t*_R_ 14 min). Fraction 1I (hexane/EtOAc = 2/1, 30.1 mg) was fractionated using Sephadex LH-20 column chromatography (*ϕ* 20 × 150 mm, CHCl_3_) to obtain fractions 2A and 2B, respectively. Fraction 2A (21.9 mg) was separated by RP-HPLC (Inertsil C8-3 5 μm, *ϕ* 10 × 250 mm; 70% CH_3_CN; flow rate 5.0 mL/min) to isolate compound **1** (0.9 mg, *t*_R_ 7 min). Fraction 2B (4.0 mg) was separated by RP-HPLC (COSMOSIL π-NAP, *ϕ* 10 × 250 mm; 80% CH_3_CN; flow rate 5.0 mL/min) to isolate compound **16** (0.7 mg, *t*_R_ 4 min). Fraction 1J (hexane/EtOAc = 2/1, 23.9 mg) was separated by RP-HPLC (COSMOSIL π-NAP, *ϕ* 10 × 250 mm; 57% CH_3_CN; flow rate 5.0 mL/min) to isolate compounds **2** (0.5 mg, *t*_R_ 6 min), **1** (0.8 mg, *t*_R_ 6.5 min), **3** (0.7 mg, *t*_R_ 7 min), **18** (0.7 mg, *t*_R_ 8 min), and **17** (0.4 mg, *t*_R_ 9 min). Fraction 1L (CHCl_3_/MeOH = 40/1, 32.7 mg) was separated by RP-HPLC (COSMOSIL Cholester, *ϕ* 10 × 250 mm; 50% CH_3_CN; flow rate 5.0 mL/min) to isolate compounds **4** (2.6 mg, *t*_R_ 10 min) and **2** (0.8 mg, *t*_R_ 11 min). The CHCl_3_ layer (711.7 mg) was fractionated by silica gel 60N column chromatography (*ϕ* 40 × 150 mm, CHCl_3_/MeOH system) to obtain fractions 3A–3F. Fraction 3B (CHCl_3_/MeOH = 80/1, 101.0 mg) was further fractionated by ODS column chromatography (*ϕ* 20 × 200 mm, H_2_O/MeOH system) to obtain fractions 4A and 4B. Fraction 4A (H_2_O/MeOH = 25/75, 66.0 mg) was separated by RP-HPLC (COSMOSIL Cholester, *ϕ* 10 × 250 mm; 48% CH_3_CN; flow rate 5.0 mL/min) to isolate compounds **6** (1.9 mg, *t*_R_ 5 min), **5** (7.2 mg, *t*_R_ 6 min), **8** with **9** (5.6 mg, *t*_R_ 7 min), **10** with **11** (5.2 mg, *t*_R_ 8 min), **7** (1.9 mg, *t*_R_ 10 min), and **4** (3.4 mg, *t*_R_ 12 min). The EtOAc layer (1.3 g) was fractionated by ODS column chromatography (*ϕ* 40 × 180 mm, H_2_O/MeOH system) to obtain fractions 5A–5D. Fraction 5A (H_2_O/MeOH = 50/50, 631.2 mg) was fractionated by RP-HPLC (COSMOSIL π-NAP, *ϕ* 10 × 250 mm; 20% CH_3_CN; flow rate 5.0 mL/min) to obtain fractions 6A–6E. Fraction 6A (74.6 mg, *t*_R_ 8 min) was separated using RP-HPLC (COSMOSIL Cholester, *ϕ* 10 × 250 mm; 20% CH_3_CN; flow rate 5.0 mL/min) to isolate compound **12** (30.9 mg, *t*_R_ 13 min). Fraction 6B (38.6 mg, *t*_R_ 10 min) was separated using RP-HPLC (COSMOSIL Cholester, *ϕ* 10 × 250 mm; 45% MeOH; flow rate 4.0 mL/min) to isolate compound **13** (19.0 mg, *t*_R_ 13 min). Fraction 6C (34.7 mg, *t*_R_ 11 min) was separated using RP-HPLC (COSMOSIL Cholester, *ϕ* 10 × 250 mm; 50% MeOH; flow rate 4.5 mL/min) to isolate compound **14** (17.0 mg, *t*_R_ 12 min). The isolated compounds were identified by comparing their spectral data with those reported in the literature.

## 3. Results and Discussion

### 3.1. Isolation of Compounds from Blumea Lacera

**Figure 1:**
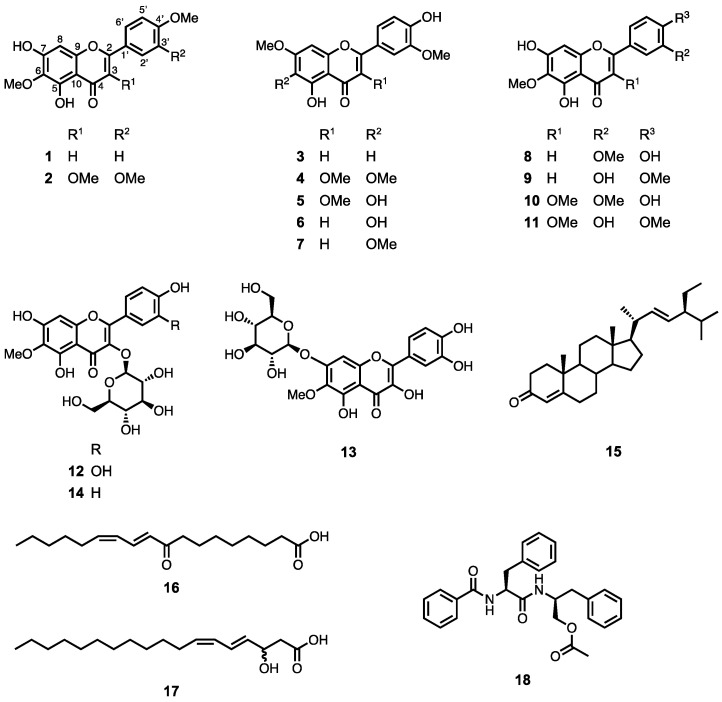
Structures of compounds **1**–**18**.

By screening Thai plants for prospective TRAIL resistance-overcoming activity, *Blumea lacera* extract was identified as a hit sample (45% inhibition at 50 μg/mL with TRAIL). The methanol (MeOH) extract of the whole *Blumea lacera* plant was partitioned using hexane, chloroform (CHCl_3_), ethyl acetate (EtOAc), and 1-butanol (BuOH). The fractionation of each layer, except the BuOH layer, was guided by the TRAIL resistance-overcoming activity test. The hexane layer was fractionated by various column chromatography methods to obtain compounds **1**–**4** and **15**–**18**. Compounds **5**–**11** were isolated from CHCl_3_. Compounds **8** and **9** (**8**:**9** = 1:1) and **10** and **11** (**10**:**11** = 1:1) were obtained as inseparable mixtures. The EtOAc layer was also fractionated to obtain compounds **12**–**14** (Figure 1). Detailed NMR and mass spectrometry analysis identified the isolated compounds as pectolinargenin (**1**) [10], bonanzin (**2**) [11], velutin (**3**) [12], chrysosplenol B (**4**) [13], chrysosplenol C (**5**) [13], 4’,5-dihydroxy-3’,7-dimethoxyflavone (**6**) [14], cirsilineol (**7**) [15], eupatilin (**8**) [16], 3-demethoxycentaureidin (**9**) [17], jaceidin (**10**) [18], centaureidin (**11**) [19], patuletin 3-*O*-β-D-glucopyranoside (**12**) [20], patulitrin (**13**) [21], 6-methoxykaempferol 3-*O*-β-D-glucopyranoside (**14**) [22], 3-oxostigmasta-4,22*E*-diene (**15**) [23], 9-oxo-10*E*,12*Z*-octadecadienoic acid (**16**) [24], 3-hydroxy-octadeca-4*E*,6*Z*-dienoic acid (**17**) [25], and aurantiamide acetate (**18**) [26].

### 3.2. TRAIL Resistance-Overcoming Activity of Isolated Compounds

**Figure 2:**
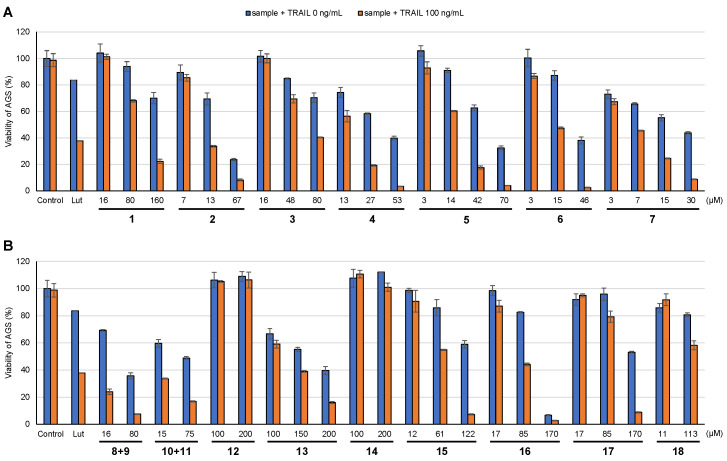
TRAIL resistance-overcoming activity. (**A**) compounds **1**–**7**, (**B**) compounds **8**-**18**. Data are presented as the mean ± SD (*n* = 3). Lut, luteolin; SD, standard deviation. Luteolin (17.5 µM) was used as a positive control.

The ability of the isolated compounds to overcome TRAIL resistance was examined using the human gastric adenocarcinoma cell line AGS. Compounds **1**–**7** and **15**–**17**, along with two mixtures of compounds (**8**/**9** and **10**/**11**), exhibited TRAIL resistance-overcoming activity. Most of the isolated flavonoids exhibited TRAIL resistance-overcoming activity (Figure 2). Bonanzin (**2**) and cirsilineol (**7**) had particularly strong TRAIL resistance-overcoming activity, with IC_50_ values of 10.7 μM and 5.9 μM in the presence of TRAIL (100 ng/mL), whereas compounds **2** and **7** had IC_50_ values of 26.1 μM and 20.7 μM without TRAIL, respectively (Figure 2). There have been no reports on the TRAIL resistance-overcoming activity of these compounds [27]. Patulitrin (**13**), a 7-*O*-glucoside of patuletin, did not exhibit strong TRAIL resistance-overcoming activity, but was cytotoxic against AGS cells, with an IC_50_ value of 165 μM. However, the other two glucosides of the flavonoids (**12** and **14**) exhibited no bioactivity at 200 μM, where it is proposed that the presence of glucose at C-3 prevents these compounds from interacting with the target. Further research is necessary to clarify these interactions.

## 4. Conclusions

Eighteen compounds, including fourteen flavonoids, were isolated from the MeOH extract of *Blumea lacera* and its partitions, guided by the TRAIL resistance-overcoming activity. Compounds **1**–**7** and **15**–**17**, along with two mixtures of compounds (**8**/**9** and **10**/**11**), exhibited TRAIL resistance-overcoming activity, whereas compound **13** was cytotoxic against AGS cells without TRAIL resistance-overcoming activity. Although the compounds have the same flavonoid skeleton, the strength of the TRAIL resistance-overcoming activity depends on the substituents. Among the isolated flavonoids, bonanzin (**2**) and cirsilineol (**7**) exhibited strong TRAIL resistance-overcoming activity, with IC_50_ values of 10.7 μM and 5.9 μM, respectively.

## Data Availability

The data that support the findings of this study are available from the corresponding authors upon reasonable request.
